# Functional Dimorphism Analysis of Sporotrophophyll Leaves and Nest Leaves of *Drynaria roosii* with Their Connected Rhizomes Based on Multi-Omics Analysis

**DOI:** 10.3390/metabo15120805

**Published:** 2025-12-18

**Authors:** Ye Cao, Yan Ren, Yanlei Han, Xiaoqing Wang, Hui Li, Yong Zeng, Xiwen Li, Ye Wang

**Affiliations:** 1 Jiangxi Key Laboratory for Sustainable Utilization of Chinese Materia Medica Resources, Institute of Traditional Chinese Medicine Health Industry, China Academy of Chinese Medical Sciences, Nanchang 330115, China; 2 Jiangxi Institute of Traditional Chinese Medicine Health Industry, Nanchang 330115, China; 3 Jiangxi Provincial Institute of Traditional Chinese Medicine, Nanchang 330046, China; 4 Institute of Chinese Materia Medica, China Academy of Chinese Medical Sciences, Beijing 100700, China; 5 School of Pharmacy, Chengdu University of Traditional Chinese Medicine, Chengdu 611137, China; 6 Institute of Medicinal Plant Development, Chinese Academy of Medical Sciences, Peking Union Medical College, Beijing 100193, China

**Keywords:** *Drynaria roosii*, sporophyll, rhizomes, multi-omics, medicinal fern

## Abstract

**Background**: *Drynaria roosii* is a typically epiphytic fern characterized by the intriguing phenomenon that nest leaves (NLs) and sporotrophophyll leaves (SLs) are metatypical, with NLs persisting in a hardened form. Few reports have concentrated on the physiological characteristics of these two leaf types and the metabolite differences in their associated rhizomes (NRs and ORs). **Methods**: A comparative analysis of the two leaf types and their connected rhizomes was conducted based on photosynthetic parameters, leaf ultrastructure, Illumina HiSeqTM 2000 transcriptome sequencing, widely targeted metabolomics, and the spatial distribution of flavonoid components. **Results**: The results indicated that SLs exhibited significant advantages in photosynthetic parameters, with a net photosynthetic rate exceeding that of NLs by 228%. A total of 7236 differentially expressed genes (DEGs) were identified between SLs and NLs, with the majority of DEGs involved in developmental processes (491 DEGs), stress response (420 DEGs), and responses to abiotic stimuli (337 DEGs). A total of 1409 components were detected and authenticated, revealing that ORs contained relatively high levels of flavonoids, quinones, tannins, alkaloids, terpenoids, and vitamins. Furthermore, the spatial distribution of flavonoid components indicated a dispersive distribution in both NRs and ORs. **Conclusions**: This comprehensive study of NLs and SLs, along with their connected rhizomes, provides vital reference for scientific cultivation management and rational harvesting prior to medicinal use.

## 1. Introduction

The cultivation of ferns requires a strict environment and daily management due to their unique traits to water and light intensity. Although both C3 photosynthesis and crassulacean acid metabolism occur in certain fern species, such as *Platycerium bifurcatum*, these adaptations enhance high water-use efficiency [1,2]. The vegetational forms and tree canopies strictly limited the population quantity of some epiphytic ferns because of the specific germination conditions of spores and vegetative growth, exacerbated by increasing anthropogenic pressures that caused degradation of local ecosystems and lighting conditions [3]. Most ferns maintain a balance between growth and reproduction, with laminar surfaces processing both reproduction and photosynthesis after a long evolution under minimal sunlight energy [4,5]. Dimorphic leaves, including cover leaves and sporotrophophyll leaves, confer a high adaptability at both structural and functional levels compared to the seed plants [6]. In epiphytes, abiotic stresses such as light and drought can lead to photoinhibition and irreversible damage to photosynthetic structures. The cover leaves create a nest-like structure that collects falling leaves and insects, serving as a valuable source of potassium and calcium, while also wrapping around the rhizomes and short roots to protect them from high temperatures and intense light [7].

*Drynaria roosii* is a special perennial epiphyte characterized by dimorphic leaves, which grow on old tree trunks, limestone, and even the roofs of old buildings, as indicated by our previous local investigations [8]. The dominant generation of *D. roosii* comprises two types of leaves: the nest leaves, which serve as sterile fronds (also called cover leaves and trophophylls), measuring approximately 10 cm in diameter, and the sporotrophophyll leaves, which range from 10 to 50 cm in length and are responsible for assimilation functions and spore generation. Early reports have shown that the nest leaves exhibit short lifespans and rapid senescence due to the accelerated decline of chlorophyll content in *Platycerium bifurcatum* [3], a species with a life history similar to that of *D. roosii*. The population spread of *D. roosii* relies on vegetative propagation via rhizomes and sexual reproduction through spores generated by the sporotrophophyll leaves. Generally, older rhizomes produce new rhizomes covered in golden tomentum, from which nest leaves gradually sprout at the surface of young rhizomes during a brief growth period. This cycle of ‘the old rhizome-young rhizome’ constitutes the vegetative propagation. The rare spores of *D. roosii* can germinate into prothallus, but the formation of sporophylls from wild prothallium is challenging under natural conditions, as the occurrence of siphonogamy requires high humidity. The abovementioned factors limits yield of wild *D. roosii* rhizomes.

The dried rhizomes of *D. roosii*, processed using hot sand, are utilized in Chinese herbal medicine for various purposes within the orthopedics department. Secondary metabolites from the rhizomes of *D. roosii* promote the proliferation of osteoblasts and the growth of chondrocytes, which is beneficial for improving the prognosis of patients with frequently occurring traumatic fractures [9,10,11,12]. Flavonoids are the primary active components contributing to these clinical efficacies, with naringin serving as the content index for the quality control of Drynariae Rhizoma in the Pharmacopoeia of the People’s Republic of China and Japan [13]. The contents of secondary metabolites fluctuate based on the botanical part, growth age, and cultivated environment. Our previous study, along with research conducted by others in China, has shown that epiphytic patterns determine the metabolite diversity of rhizomes, with those on tree trunks exhibiting higher flavonoid components [8,14]. Recent physiological and differential protein expression analyses have revealed that calcium stress may affect the secondary metabolism of *D. roosii* rhizomes because of the natural distribution environment in China’s karst areas [15,16,17]. Artificial cultivation in greenhouses, supplemented with pine bark and stringent management, can generate high-content rhizomes of *D. roosii* compared with wild resources [18]. Furthermore, UV irradiation during the growth process also enhances the contents of naringin and neoeriocitrin by the up-regulating synthetic genes, such as *PAL*, *C3′H*, and *HCT* [19]. Additionally, the growth period influences the accumulation of secondary metabolites, with naringin predominantly enriched in young rhizomes, while older rhizomes contain higher levels of neoeriocitrin [20]. The large-scale cultivation of *D. roosii* holds a promising perspective in China, because various Chinese patented medicines and the high annual demand associated with the aging population in certain Asian countries [8]. The published research is often concerned about the responding variation in rhizomes under different experimental conditions. Therefore, it is essential to investigate the growth of leaves (both nest leaves and sporotrophophyll leaves) and the metabolites of their connected rhizomes.

This study was designed to test the hypothesis that the functional differentiation between two types of sporophylls in *D. roosii* systematically influences metabolic accumulation patterns in their connected rhizomes. We employed a multi-omics approach integrating photosynthetic profiles, leaf ultrastructure, transcriptomic profiling, and widely targeted metabolomics. Our specific objectives were to (1) characterize the photosynthetic and transcriptional divergence between two types of sporophylls, and (2) determine the differences in metabolite accumulation between the rhizomes connected to these different leaf types. This comprehensive comparative research aims to provide a rational guide for the yield management of leaves and the optimal harvesting of fresh rhizomes in the large-scale cultivation of *D. roosii*.

## 2. Materials and Methods

### 2.1. Experimental Design

Wild rhizomes of *D. roosii*, containing nest leaves (with the wild sporotrophophyll leaves removed), were collected from Huaqiao Country in Dexing City, Jiangxi Province, China (28.95 N, 117.76 E), and cultivated in the climate chamber at the Institute of Traditional Chinese Medicine Health Industry, China Academy of Chinese Medical Sciences starting in 2023. The fresh leaves that germinated in 2025 were utilized for the detection of photosynthetic parameters and leaf ultrastructure, which were categorized into nest leaves (NLs) and sporotrophophyll leaves (SLs). Fresh rhizomes and leaves were frozen in liquid nitrogen and stored at −80 °C for transcriptome and metabolomics analysis. These rhizomes were classified into two groups: new rhizomes (NRs) with gold tomentum connecting fresh nest leaves, and old rhizomes (ORs), where the gold tomentum has been covered by NL and transformed into a black form.

### 2.2. Detection of Photosynthetic Parameters and Leaves’ Ultrastructure Between NLs and SLs of D. roosii

The photosynthetic capacity of *D. roosii* is typically assessed by evaluating the combined performance of stomatal and chloroplast status. Therefore, we conducted an analysis of photosynthetic light response curves, photosynthetic CO_2_ response curves, gas exchange, and fluorescence parameters to compare the potential difference in photosynthesis between NLs and SLs. The ultrastructure of the leaves provides insights into the actual characteristics of chloroplasts, stomata, and other organelles in the two types of sporophylls of *D. roosii*.

#### 2.2.1. The Measurement of Photosynthetic Light/CO_2_ Response Curves of NLs and SLs

Mature NLs and SLs were employed to obtain the photosynthetic light response curve and the photosynthetic CO_2_ response curve with the help of a LI-6800 portable photosynthesizer (Li-Cor, Lincoln, NE, USA) within a 6800-01A fluorescent chamber. Measurements were conducted at 27 °C with 65% relative humidity of air around the leaves, using a flow speed of 500 µmol/s and fan revolutions of 10,000 r/min.

The CO_2_ concentration was fixed at 400 μmol/mol for the detection of the photosynthetic light response curve combined with light intensity (Qin values) as follows: 1800, 1500, 1200, 900, 600, 300, 200, 150, 100, 70, 30, and 0 μmol/(m^2^·s). The minimum and maximum waiting times were set to 60 s and 200 s, respectively. The photosynthetic light curve was generated using photosynthesis-fitting software (version 7z.1), which considered the net photosynthetic rate and light intensity values. Meanwhile, four evaluation parameters were calculated based on the response curve: light compensation point (LCP), light saturation point (LSP), maximum photosynthetic rate (MPR), apparent quantum efficiency (AQE), dark respiration rate (DSR), fitting residuals (FR), and determination coefficients (R2).

Furthermore, the light intensity value was set to 49 μmol/(m^2^·s) based on the long-term actual environment of the climate chamber for better suitability for *D. roosii*. The photosynthetic CO_2_ response curve was scanned using concentrations of 400, 300, 200, 100, 50, 3, 400, 600, 800, 1000, 1200, 1500, 1800, and 2000 μmol/mol. All other parameters were consistent with those used in the photosynthetic light response curve. The fitting evaluation parameters derived from the fitting curves included the CO_2_ compensation point (CCP), CO_2_ saturation point (CSP), MPR, AQE, DSR, FR, and R^2^. A minimum of 3–5 individuals from each type of sporophyll were analyzed and the average values were employed for the final calculations and comparative analysis.

#### 2.2.2. Record of Gas Exchange and Fluorescence Parameters of NLs and SLs

The gas exchange and fluorescence parameters were obtained based on the parameter settings analogous to the response curves. The gas exchange and fluorescence-related parameters under light included the net photosynthetic rate (Pn), transpiration rate (E), total conductance to water vapor (gtw), total conductance to CO_2_ (gtc), stomatal conductance to water vapor (gsw), stomatal conductance to boundary layer water vapor (gbw), intercellular CO_2_ concentration (Ci), vapor pressure deficit at the leaf surface (VPD), steady state fluorescence under light (Fs), maximum fluorescence under light (Fm″), actual quantum yield of photosystem II photochemistry (PhiPS2), electron transport rate (ETR), quantum efficiency of CO_2_ assimilation (PhiCO_2_), non-photochemical quenching coefficient (NPQ), and the difference between maximum fluorescence and minimum fluorescence under light (Fv′). Four fluorescence-related parameters after dark adaptation were obtained including minimum initial fluorescence (Fo), maximum fluorescence after dark adaption (Fm), maximum photochemical efficiency of PSII reaction centers when fully open under dark adaptation (Fv/Fm), and net photosynthetic rate under dark adaptation (Adark). A total of five individuals of each type of sporophyll were assessed, and the averaged values were utilized for the final comparative analysis.

#### 2.2.3. The Scan of Stomata and Ultrastructure of NL and SL

Two types of sporophylls were cut into small pieces with sizes of 0.5 × 0.5 cm, avoiding the leaf midrib. These samples were fixed in 4% glutaraldehyde buffer solution at 4 °C for 2 h, then rinsed three times (15 min each) with 0.1 M phosphate buffer (pH 7.4). Subsequently, these small pieces were post-fixed in a mixed 1% osmic acid solution prepared with 0.1 M phosphate buffer (PH = 7.4) for 2 h at room temperature, protected from light. The second fixation of the sporophylls was followed by three washes with 0.1 M phosphate-buffered solution (PH = 7.4, 15 min each time).

The post-fixed sporophylls were dehydrated using a series of ethanol solutions at varying concentrations (30%, 50%, 70%, 80%, 90%, 95%, 100%, and 100%, respectively) for 15 min each time, followed by immersion in isopentyl acetate for 15 min. The prepared samples were then dried using the K850 critical point drying (Quorum, Brighton, UK) and subjected to electrical conductivity treatment using the MC1000 ion sputter coater (Hitachi, Hitachi City, Japan). Finally, the sample tables were scanned with the SU8100 scanning electron microscope (Hitachi, Japan) to compare the status and density of somas on the surfaces of the two types of sporophylls.

The post-fixed sporophylls underwent dehydration through a series of ethanol concentrations (30%, 50%, 70%, 80%, 95%, 100%, and 100%, each for 60 min), followed by a sequential treatment with ethanol/acetone mixture of 3:1 for 30 min, 1:1 for 30 min, and 1:3 for 30 min, concluding with pure acetone for 60 min. Subsequently, the dehydrated samples were infiltrated with acetone/812 resin in a ratio of 3:1 for 2 h, followed by a ratio of 1:1 for 8 h and 1:3 for 2 h, before being immersed in pure 812 resin for 5 h under 37 °C. The embedding plates containing pure 812 resin and the dehydrated samples were placed in a drying oven at 37 °C for 8 h and subsequently heated at 60 °C for 48 h. The resulting resin blocks were cut into slices using ultra-microtomes (version UC7, Leica, Wetzlar, Germany), and these slices were stained for 8 min with a 2% saturated alcoholic solution of uranium acetate, protected from light. The stained slices were then washed three times with 70% ethanol and water, followed by a secondary staining for 8 min with a 2.6% lead citrate solution, and then washed three times with ultrapure water. Finally, the intact and well-stained slices were examined using an HT7700 transmission electron microscope (Hitachi, Japan) to analyze the ultrastructure of specific organelles.

### 2.3. Transcriptome Measurement of NLs and SLs of D. roosii

Two types of sporophylls from *D. roosii* were extracted for total RNA using RNAprep Pure Plant Hit (TIANGEN, Beijing, China) from a freezer at −80 °C. The extracted RNA concentration and integrity were determined by a 2100 Bioanalyzer (Agilent Technologies, Santa Clara, CA, USA). High-quality RNA was further isolated and enriched for messenger RNA (mRNA) for the synthesis of cDNA through the addition of Oligo (dT) and fragmentation. The resulting cDNA, prior to fragment selection and PCR enrichment, was mixed with polyA tails and adapters for library construction. The library quality was evaluated using the Q-PCR method, ensuring an effective concentration greater than 2 nM. A total of six cDNA libraries were analyzed to obtain gene sequences using the Illumina HiSeqTM 2000 (Illumina, San Diego, CA, USA).

These raw sequences were processed to obtain high-quality reads by removing inferior paired reads with more than 10% N content and partial reads where more than 50% of bases had a quality score of Q ≤ 20. Reference transcripts were assembled using Trinity (V2.4.0) after selecting clean reads [21]. Differentially expressed genes (DEGs) were identified using DESeq2 software (R4.1.2) from those reads without normalization between NLs and SLs [22], with selection criteria based on two profiles: false discovery rate (FDR) < 0.05 and |log_2_Fold Change| ≥ 1. The expression levels of DEGs were calculated using the kilobase of transcript per million fragments mapped (FPKM), with high-quality reads normalized prior to analysis. These DEGs underwent annotation analysis based on the Kyoto Encyclopedia of Genes and Genomes (KEGG) and the Gene Ontology (GO) database to interpret the potential functions of genes involved in metabolites synthesis and other functional profiles in cellular components, biological processes, and molecular functions.

### 2.4. Measurement and Analysis of Widely Targeted Metabolomics Between Connected Rhizomes of NRs and ORs of D. roosii

The extraction and detection of the rhizomes of *D. roosii* was conducted following the methods in our previous study [8,23]. In brief, 0.05 g of rhizome powder from these freeze-dried samples was extracted using 1.2 mL of 70% methanol solution under vortex oscillation, with a L-2-chlorophenylalanine (purity 98%, CAS 10361089-3) included as the internal standard. The resulting supernatant, obtained after centrifugation, was filtered through a microporous membrane for final analysis via ultra-performance liquid chromatography–electrospray ionization–mass spectrometry (UPLC-ESI-MS/MS).

The extraction solution of rhizomes was separated and qualitatively analyzed using a UPLC-ESI-MS/MS system (UPLC, ExionLCTM AD, MS/MS, Applied Biosystems QTRAP 6500, Foster City, CA, USA). The UPLC separation was followed as 5–95% B from 0 min to 9 min, 95% B from 9 to 10 min, 95–5% B from 10 to 11.10 min, and 5% B from 11.10 min to 14 min, of which B was acetonitrile containing 0.1% formic acid and the adjustment of the mobile phase was performed by water containing 0.1% formic acid. The flow rate, injection volume, and column temperature were set at 0.35 mL/min, 2 μL, and 40 °C, respectively. The mass spectrometry method utilized a targeted scan acquisition cycle of 1.0 s, with individual dwell times ranging from a minimum of 2 ms to a maximum of 50 ms. Electrospray ionization was performed at 550 °C, with voltage configurations established at +5500 V for positive ion mode and −4500 V for negative ion mode. Gas parameters were meticulously controlled, with gas I set at 50 psi, gas II at 60 psi, and curtain gas maintained at 25 psi. Collision-induced dissociation operated with a nitrogen gas flow rate of 2.00 L/min. The entrance potential was set at +10.00 V (positive mode) and −10.00 V (negative mode), while the collision cell exit potential was +5.00 V and −11.00 V for positive and negative modes, respectively. Quantitative analysis leveraged triple quadrupole (QQQ) scanning in multiple reaction monitoring (MRM) mode at medium sensitivity. MRM transition pairs were established through systematic optimization of declustering potential (DP) and collision energy (CE) parameters. Continuous monitoring of specific precursor-product ion transitions was implemented during defined experimental intervals to target analyte-specific detection.

Qualitative and quantitative mass spectrometry analyses were varied out using datasets from MRM and the MetWare database. The mass dataset analysis was conducted using Analysis 1.6.3 software (AB Sciex, Boston, MA, USA).

### 2.5. Measurement and Analysis of Spatial Distribution of Flavonoid Components Between Connected Rhizomes of NRs and ORs of D. roosii

Fresh rhizomes, after being refrigerated at −80 °C, were sliced into 50 μm thin sections using a cryostat and subsequently placed on the surface of indium tin oxide glass. These thin sections were then sprayed with a matrix solution comprising 10 mg/mL of 9-AA in a 1:1 mixture of methanol and water (*v*/*v*) to enhance ionization efficiency. Furthermore, they were analyzed using a solid-state laser (λ = 343 nm) operating at a repetition rate of 2000 Hz in negative ion mode. The measurement rate in full scan mode (scan range *m*/*z* 150–1400) was set to 1.5 s/pixel, achieving a mass resolution of 70,000 and a mass accuracy within 2 ppm at *m*/*z* 200. The spatial resolution for the mass spectrometry imaging analysis was 20 μm. Imaging analysis of the molecular weights corresponding to 73 flavonoids was performed using Shimadzu’s mass spectrometry imaging data analysis software, IMAGREVEAL MS (Version 1.21 0.1130).

## 3. Results

### 3.1. The Morphological Variation in NLs and SLs of D. roosii During the Whole Growth Cycle

The expansion of the *D. roosii* population relies on two primary modes of reproduction: sexual reproduction through mature spores and asexual reproduction via rhizomes. The collected samples used in the present study were harvested from original seedlings through asexual reproduction. The rhizomes were cultivated in a flower plot in which sporotrophophyll leaves began to germinate on the older rhizomes (Figure 1① and ③). The generation of NLs is often associated with young rhizomes characterized by a gold tomentum (Figure 1④ and ⑥). Partial SLs can produce spores, and the mature sporangia serve as the core propagation materials for seedling production. Green NLs typically die within approximately 20 days and cover the surface of the rhizomes, contrasting with SLs, which are easily deciduous after dry rotting.

### 3.2. The Comparison of Photosynthetic Parameters and Leaves’ Ultrastructure Between NLs and SLs of D. roosii

Significant photosynthetic differences emerged between leaf types under identical growth conditions (Table 1). Three respiration parameters exhibited extremely significant differences (*p* < 0.01). Specifically, SLs demonstrated a higher net photosynthetic rate (Pn) compared to NLs (SLs: 1.0039 ± 0.2687 µmol·m^−2^ s^−1^; NLs: 0.3063 ± 0.1068 µmol·m^−2^ s^−1^) and a greater vapor pressure deficit at the leaf surface (SLs: 1.6554 ± 0.0132 kPa; NLs: 1.2694 ± 0.0229 kPa), while exhibiting a lower stomatal conductance to boundary layer water vapor (VPD) than NLs (SLs: 3.0215 ± 0.005 mol·m^−2^·s^−1^; NLs: 3.0508 ± 0.0014 mol·m^−2^·s^−1^). In contrast, NLs exhibited significantly higher steady state fluorescence under light (Fs, 336.4477 ± 15.4555) and a greater difference between maximum fluorescence and minimum fluorescence under light (Fv′, 1.0009 ± 0.0001) than those of SLs (Fs = 256.2163 ± 24.5645; Fv′ = 0.7865 ± 0.0179). However, SLs had a significantly higher actual quantum yield of photosystem II photochemistry (0.7175 ± 0.0279) compared to NLs (0.6591 ± 0.021). No significant differences were observed in the remaining 13 respiration and fluorescence parameters between SLs and NLs of *D. roosii*.

Furthermore, two important response curves, namely the photosynthetic light response curve (PLSC) and the photosynthetic CO_2_ response curve (PCRC), were calculated to obtain the fitting parameters for SLs and NLs (Table 2). The fitting results of the PLSC indicated that SLs exhibited higher maximum net photosynthetic rates (Pnmax), light saturation points (LSPs), and apparent quantum yields (AQYs) compared to NLs, with a determination coefficient exceeding 0.8 and low fitting residuals (<1.7085). Specifically, the Pnmax value for SLs was found to be 5.96 times greater than that of NLs. In contrast, NLs demonstrated a higher light compensation point (16.8850 µmol·m^−2^·s^−1^) compared to SLs (11.5740 µmol·m^−2^·s^−1^). The PCRC analysis, particularly for SLs, showed poor performance characterized by high fitting residuals and a low determination coefficient. Nevertheless, an obvious difference remained between SLs and NLs, with SLs exhibiting higher values in dark respiration rate, maximum net photosynthetic rate, and saturation intercellular CO_2_ concentration. Furthermore, NLs had a higher CO_2_ compensation point than SLs, with fitting residuals of 0.6672 and a determination coefficient of 0.9110.

**Figure 2:**
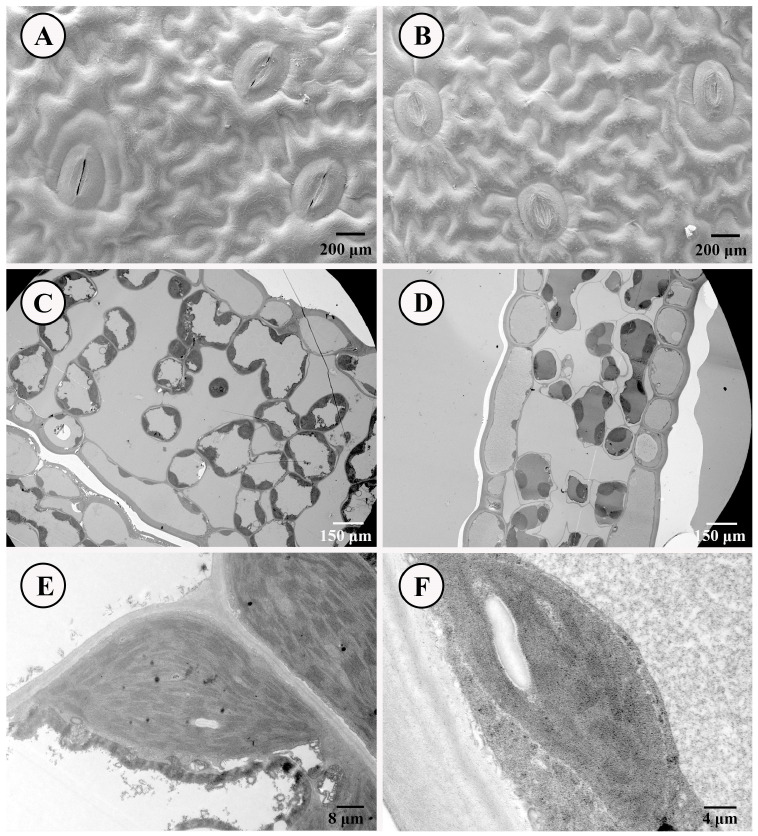
Stoma and leaves’ ultrastructure of SLs and NLs on *D. roosii*. ((**A**)—stoma status of SLs, (**B**)—stoma status of NLs, (**C**)—the distribution of chloroplast in mesophyll tissue of SLs, (**D**)—the distribution of chloroplast in mesophyll tissue of NLs, (**E**)—grana thylakoids of chloroplast of SLs, (**F**)—grana thylakoids of chloroplast of NLs).

The scanning electron microscope and transmission electron microscope were employed to compare the differences in stomatal structure and chloroplast status between SLs and NLs of *D. roosii*. The comparative results indicated that most stoma in SLs were patulous, whereas those in NLs were closed (Figure 2A,B). Both types of stomata belong to the anomocytic category, characterized by 3–5 upper epidermis cells. These findings were consistent with respiratory measurements, which showed that SLs exhibited high Pn, VPD, transpiration rates, stomatal conductance to water vapor, total conductance to water vapor, and total conductance to CO_2_, while displaying lower intercellular CO_2_ concentrations. The upper epidermis of both leaf types consisted of a layer of closely arranged cells with minimal presence of chloroplasts. SLs contained a greater number of chloroplasts within the mesophyll tissue compared with NLs (Figure 2C,D), and the individual chloroplasts in SLs possessed more grana thylakoids (Figure 2E,F). Partial chloroplasts contained starch grains within these grana thylakoids.

### 3.3. Comparison Results of Differential Expression Gene Networks Between NLs and SLs of D. roosii

A total of 40,719,400 to 41,819,434 raw reads and 6,107,910,000 to 6,272,915,100 raw bases were obtained after sequencing. Following this, 40,429,050 to 41,538,090 clean reads and 5,913,912,679 to 6,192,381,618 clean bases were acquired, reflecting a clean ratio exceeding 99.29%. High Q20 (>98.29%) and Q30 (>94.94%) indicated a low error rate during the sequencing process (Table S1). Ultimately, 90.57% to 92.03% of reads were mapped for differential expression and annotation analysis (Table S2). Detailed profiles, including expression level, statistical parameters, and the annotation results GO and KEGG for each gene, are presented in Table S3. Table S3 provides comprehensive transcriptomic data documenting expression levels, statistical parameters, and functional annotations GO and KEGG for all identified genes. These data provide the molecular foundation for understanding the functional dimorphism between sporotrophophyll and nest leaves in *D. roosii*.

**Figure 3:**
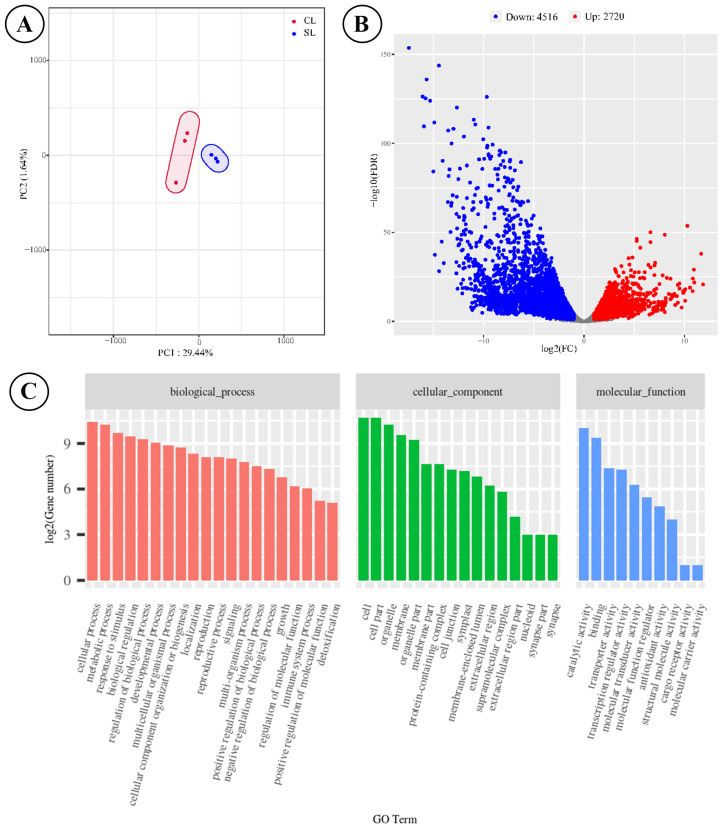
Analytical results of transcriptome sequencing of SLs and NLs on *D. roosii*. ((**A**)—score plot of principal component analysis based on expressed genes, (**B**)—volcano plot of DEGs, (**C**)—GO annotations for DEGs).

The score plot of PCA based on these expressed genes revealed a significant difference between the two types of fronds, as evidenced by the two distinct cluster circles (Figure 3A). A total of 7236 DEGs were identified between SLs and NLs, with NLs exhibiting 2720 up-regulated genes and 4516 down-regulated genes (Figure 3B and Table S3). We obtained potential GO annotations for 1960 DEGs categorized into three domains: biological processes, cellular components, and molecular functions (Figure 3C). Among these DEGs of NL, there were 1346 down-regulated and 614 up-regulated genes within the GO annotation profiles. The enrichment analysis indicated that the majority of DEGs participated in developmental processes (491 DEGs), responses to stress (420 DEGs), and responses to abiotic stimulus (337 DEGs). The annotation results demonstrated the potential function difference in the two types of leaves after long-term evolution. KEGG enrichment analysis of the 1723 differentially expressed genes identified the phenylpropanoid biosynthesis pathway as the most significantly enriched metabolic node (*p* < 0.001), representing the core biosynthetic route for flavonoid precursor generation. Our transcriptomic analysis revealed coordinated up-regulation of key genes throughout this pathway in sporotrophophyll leaves, including one phenylalanine ammonialyase (PAL), one 4-coumarate-CoA ligase (4CL), and two cinnamoyl-CoA reductase (CCR) genes. This coordinated transcriptional activation establishes an enhanced flux capacity through the phenylpropanoid pathway, correlating with the elevated levels of flavonoids detected in SL-connected rhizomes.

### 3.4. Metabolite Variation Between Connected Rhizomes of NLs and SLs of D. roosii

**Figure 4:**
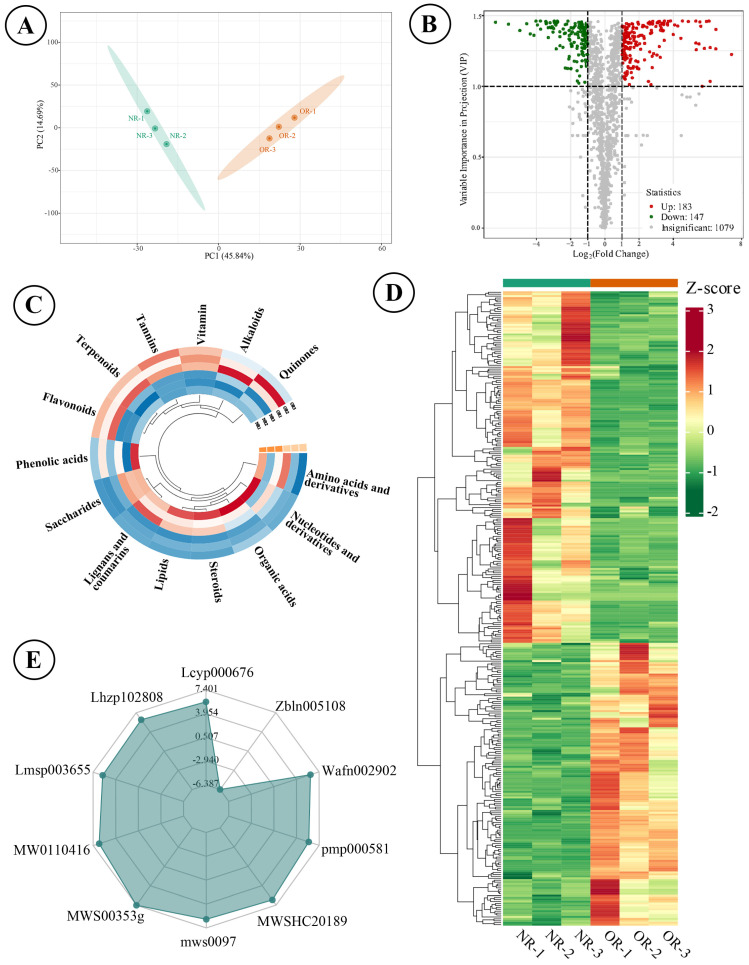
Analytical results of wild target metabolomics of ORs and NRs on *D. roosii*. ((**A**)—score plot of principal component analysis based on detected metabolites, (**B**)—volcano plot of differential metabolites, (**C**)—circle cluster heatmap of 14 categories of metabolites, (**D**)—cluster heatmap of significantly different metabolites, (**E**)—radar plot of top 10 significantly different metabolites).

**Figure 5:**
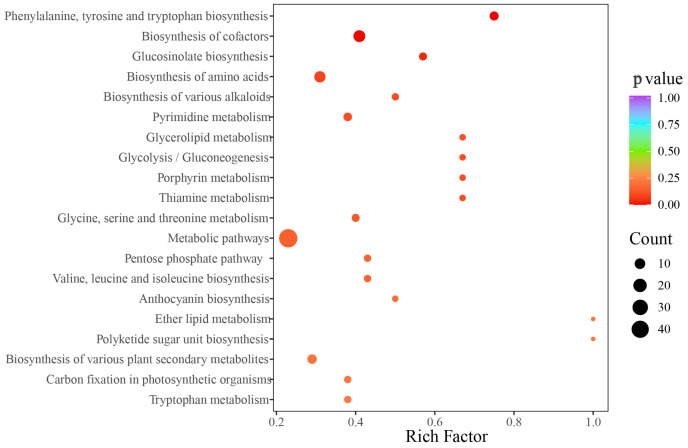
KEGG enrichment analysis result of wild target metabolomics ORs and NRs on *D. roosii*.

A total of 1409 components were detected and authenticated in the two types of rhizomes, categorized into 15 groups, including amino acid and their derivatives (173), nucleotides and their derivatives (47), organic acids (70), steroids (11), lipids (173), lignans and coumarins (91), saccharides (65), phenolic acids (134), flavonoids (279), terpenoids (102), tannins (23), vitamins (16), alkaloids (105), quinones (12), and others (108). The qualitative analysis revealed that the predominant metabolites were flavonoids and amino acids (and their derivatives). The score plot indicated that the two types of rhizomes could be distinctly separated into two clusters using the first two principal components, which explained 60.53% of the variables’ information (Figure 4A). A total of 330 differential metabolites were identified, of which 183 metabolites were up-regulated in ORs compared with NRs (Figure 4B). The accumulated content across 14 categories showed that ORs contained relatively high levels of flavonoids, quinones, tannins, alkaloids, terpenoids, and vitamins, while the other eight categories exhibited higher content in NRs (Figure 4C). The comparison of the relative content of all differential metabolites was conducted using a cluster heatmap, with the content variation illustrated in Figure 4D. The top 10 metabolites identified were octanoyl arabinosylglucoside (Wafn002902), apigenin-7-O-(6″-acetyl)glucoside (pmp000581), epipinoresinol (MWSHC20189), 6-hydroxyhexanoic acid (mws0972), α-methyl-DL-phenylalanine (MWS00353g), Val-Phe (MW0110416), dehydrodiconiferyl alcohol-γ′-O-glucoside (Lmsp003655), sesamin (Lhzp102808), and methylesculin (Lcyp000676), which spanned four categories (lignans and coumarins, saccharides, organic acids, amino acids and their derivatives) (Figure 4E). Enrichment analysis pointed to these differential metabolites as predominantly involved in metabolic pathways, biosynthesis of cofactors, and biosynthesis of amino acids with abundant enrichment of metabolites, as well as ether lipid metabolites and polyketide sugar unit biosynthesis being notably high (Figure 5).

**Figure 6:**
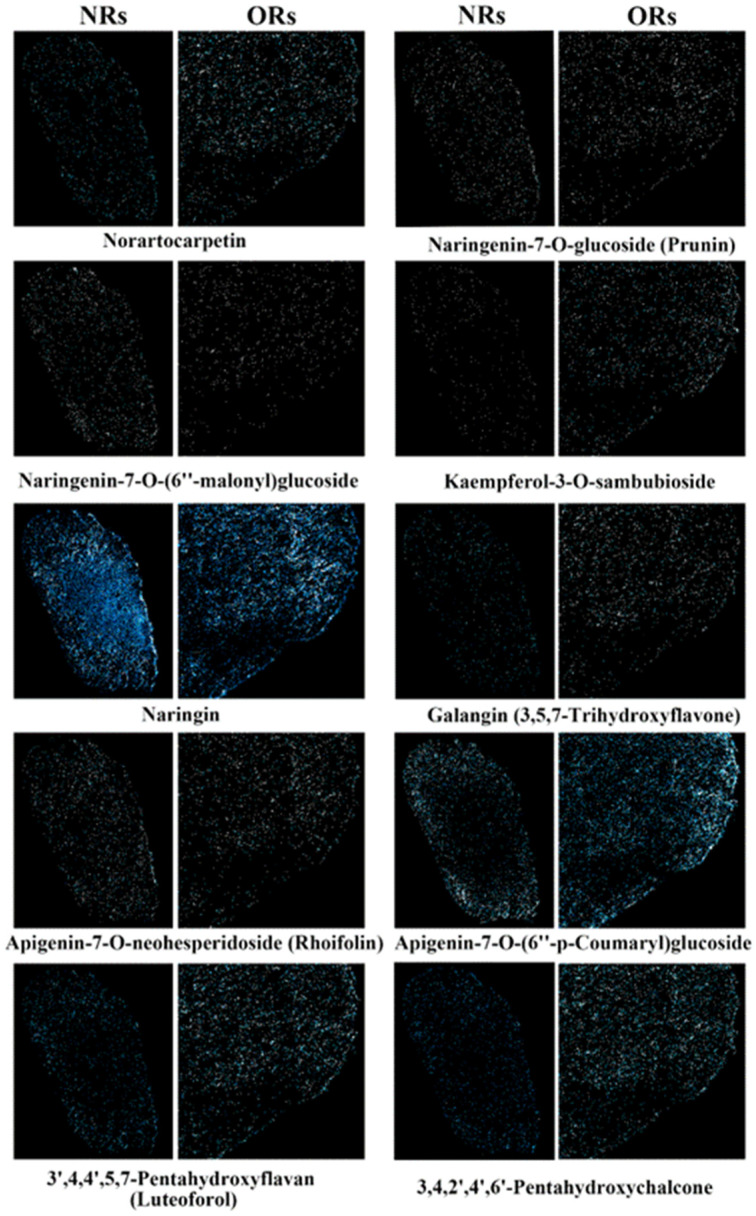
The spatial distribution of the main 10 flavonoid components in ORs and NRs of *D. roosii*. (the blue points mean the targeted metabolites and high imaging intensity means higher relative contents).

Both NRs and ORs exhibited tomentum on their surfaces, which was burned rapidly before being used in clinical patent medicine in China. We further conducted an analysis of the spatial distribution of flavonoid components within the cross-section of the rhizomes. A total of forty-two flavonoids were detected, resulting in improved imaging outcomes. The top ten flavonoid components in NRs and ORs are presented in Figure 6. These comparative results indicated that naringin was the most abundant component, serving as the assessment index for herbal medicine in the Chinese Pharmacopeia, with no significant difference in terms of the imaging results between NRs and ORs. Norartocarpetin, kaempferol-3-O-sambubioside, galangin, luteoforol, and 3,4,2′,4′,6′-pentahydroxychalcone demonstrated higher content in ORs with respect to the imaging performance. All targeted flavonoid components exhibited a dispersive distribution in these rhizomes even though the tomentum had not been removed. Thus, we speculated that the removal of tomentum did not affect the main flavonoid components, suggesting that traditional processing methods are rational in the actual production of crude herbal materials. Additionally, 64 other flavonoid components exhibited poor imaging performance between the two rhizomes and detailed comparison plots are summarized in Figure S1; there was no obvious difference between the two types of rhizomes due to low abundance.

## 4. Discussion

### 4.1. The Structure Difference in Stomata and Ultrastructure Between SLs and NLs of D. roosii Determined the Variations in Photosynthetic Ability

There are over 700 fern species worldwide with potential medicinal uses derived from their whole plants or rhizomes. Among these, Drynariae Rhizoma, sourced from the dried rhizome of *D. roosii*, has emerged as a focal point in the scientific research of fern plants due to its flavonoid components [24]. *D. roosii* was an intriguing epiphytic species that thrives on the trunks of old trees or on karst rock formations, and possessed persistent NLs with protective functions from the high temperature stress and drought stress under specific conditions. This unique growth pattern contributes to many of the world’s centers of plant diversity and is a significant factor in the global latitudinal diversity gradient for plants [25]. However, there are few reports focusing on the physiological variations in NLs when they maintain a fresh and green status, as the growth period in the natural condition is notably short.

While ferns generally exhibit more limited capacity for water-use efficiency (WUE) enhancement compared to seed plants, a key trait for mitigating drought stress by balancing water loss and CO_2_ diffusion through stomata [26,27], SLs of *D. roosii* display greater WUE. This advantage reflects an integrated adaptation involving stomatal behavior and photosynthetic performance, consistent with the natural growth patterns: NLs serve protective functions (short growth periods, <30 days), whereas SLs act as photosynthetic organs, persisting over 6 months under suitable conditions. Mechanistically, differential stomatal regulation underlies this functional divergence. Most stomata of NLs were closed or only slightly open (Figure 2), aligning with their growth under shaded conditions caused by the overlying long SLs. These variations in photosynthesis were consistent with *P. bifurcatum*, which demonstrated that SLs exhibited a stronger response of the photosynthetic apparatus to photoinhibition compared with NLs under the same conditions [6]. SLs had significantly higher PhiPS2 values than NLs, indicating that these parameters are sensitive to environmental fluctuation [28,29,30], particularly regarding light conditions and soil moisture, where minor variations in the two factors determine the regeneration and dormancy of *D. roosii*. Collectively, stomatal control, photosynthetic capacity, and light-use efficiency underscored the functional specialization of SLs as the primary photosynthetic organs in *D. roosii*. These traits reflected long-term evolutionary adaptations for improved suitability under strict and fickle climatic and epiphytic patterns among epiphytic ferns with dimorphic leaves.

The AQP calculated from PLSC reflected the transformation efficacy under weak light conditions [31]. SLs exhibited AQY values exceeding 179% compared to NLs, aligning with the growth performance of SLs across varying light intensity gradients. Notably, we observed an interesting variation that NLs transformed into SL-like shapes without elongated petioles after being subjected to stable light conditions (34 µmol·m^−2^·s^−1^) and sufficient moisture. However, these variant leaves, resembling SLs, maintained a similar growth period to that of normal NLs, but they no longer covered the rhizomes, despite remaining persistent after drying rot on the ORs. Pnmax and LSP emerged as critical parameters for evaluating adaptive capacity under stress conditions [31], indicating that SLs possess a strong potential for resilience in the face of drought or high-light stress. The PCRC further demonstrated that SLs have photosynthetic advantages, as evidenced by high SIC and CCP values. These comparative results were consistent with analytical transcriptomic data, which indicated that most DEGs were involved in developmental processes, stress responses, and reaction to abiotic stimuli. SLs have potential for spore generation, while NLs tend to deteriorate quickly, even when they assume an SL-like shape. The morphological variation in sporophylls is particularly intriguing, especially in *D. roosii*, which are vital evidence for species evolution and trade-offs between fertile–sterile dimorphy [5]. The SLs with the ability to produce spores and persistent NLs are widespread in natural environments. However, our field investigations did not reveal any instances of SL-like shape variation. Furthermore, we observed significant differences between SLs, with only a few capable of generating spores, despite conducting water and light stress experiments, which warrants further long-term and gradient studies. The combination stress condition may be contributed to SLs capable of spore generation. High-yield spore investigations should receive increased attention, as the propagation of seedlings using mature spores represents an effective protocol with low production costs. Controlled factorial stress experiments could help disentangle the environmental triggers of sporogenesis, which would provide vital contributions for the planting factory. This investigation for revealing the contributing factors of spore generation in ferns would provide an important guide for other medicinal ferns, such as *Cibotium barometz* and *Cyrtomium fortune*, which are frequently used herbs whose rhizomes are medicinal parts in China.

### 4.2. The Rational Harvesting of Rhizomes Was Vital for High-Effective Clinical Usage

The optimal harvesting time and the botanical parts significantly influence the yield and content of targeted metabolites. Most seed plants require a long growth period for medicinal purposes, particularly those medicinal plants whose roots and rhizomes are utilized for herbal medicines. The saponin content of *Paris polyphylla* var. *yunnanensis* reached its highest values at the eighth year whereas it decreased after the growth year [32]. In addition, key secondary metabolites in stem or leaf-type herbal medicines also exhibited fluctuations throughout different months of the same year [33]. Our previous study investigated the variation in metabolites in *D. roosii* across different epiphytic patterns, revealing that rhizomes in rock tunnels contained high levels of alkaloids and amino acids and their derivatives, while tree trunks were primarily associated with flavonoids and other metabolites [13]. *D. roosii* is widely distributed in the southern provinces of China, where high humidity and a warm climate contribute to prolonged growth periods [16]. The growth pattern of *D. roosii* rhizomes is analogous to that of Zingiber officinale, which also exhibits the extension of young rhizome branches within the same year [34]. Notably, old rhizomes of *D. roosii* maintain excellent vitality and asexual reproductive capacity, as demonstrated in our cultivation investigation, where old rhizomes generated new rhizomes and SLs within 20 days. The wild resources of *D. roosii* remain the primary source of crude materials of herbal medicine where NRs and ORs are indiscriminately harvested. Our cultivation study found that NRs exhibited a higher planting percentage (50%) and better growth performance compared to ORs (8.33%). Consequently, we propose a novel cultivation and harvesting mode in which NRs are utilized as raw materials for asexual reproduction while ORs are collected for medicinal usage in the artificial base, simultaneously considering the high content flavonoids of ORs (Figure 4C).

The homogeneous distribution of flavonoids throughout the rhizome tissues of *D. roosii*, with no significant variation between phloem and xylem, stands in marked contrast to the tissue-specific localization patterns typically observed in the roots or rhizomes of many other medicinal plants [34,35,36,37,38]. In comparison, monoterpene glycosides and paeonol glycosides from *Paeonia suffruticosa* and *P. lactiflora* exhibit spatial differences among cork, phloem, xylem rays, cambium, and rays [39]. Similarly, the localization patterns of ginsenosides show significant variation across different tissue sections of several *Panax* species, with the same components exhibiting differential performance in identical locations of tissue sections among different species, even within the same genus [40]. This distribution pattern in *D. roosii* may reflect the evolutionarily primitive status of ferns among vascular plants, suggesting that tissue-specific metabolite compartmentalization became increasingly specialized during the evolution of seed plants. This finding provides scientific justification for traditional processing methods that remove only the external tomentum, as this processing method does not affect the variation in the content of the flavonoid components.

*D. roosii* is a typical epiphytic fern whose growth and development are highly susceptible to environmental changes. Present studies by our research team have shown that prolonged water deficiency, intense light, and low light severely inhibit plant growth and even population expansion. The divergent evolution of its two types of sporophylls ensures the fern’s development under extreme environmental conditions. This differentiation in leaf types allows *D. roosii* to produce spores under strong light while accumulating photosynthetic products under weak light, thereby promoting biomass accumulation in the rhizome.

## 5. Conclusions

This study reveals a coordinated functional specialization in *D. roosii*, where SLs and NLs have evolved distinct physiological roles. Our integrated analysis demonstrates that SLs are the primary organs, exhibiting a significantly higher net photosynthetic rate compared to NLs, which demonstrated only minor synthesis capability during a short growth period. More importantly, the comparison of connected rhizomes indicated that ORs connected to SLs represent the optimal harvesting section for medicinal usage due to their high flavonoid contents. These findings provide a scientific basis for rational harvesting that target ORs for medicinal use while preserving NRs for sustainable propagation. Our integrated multi-omics approach offers both a systematic framework for understanding functional ecology and practical foundations for the sustainable cultivation of *D. roosii*.

## Figures and Tables

**Figure 1:**
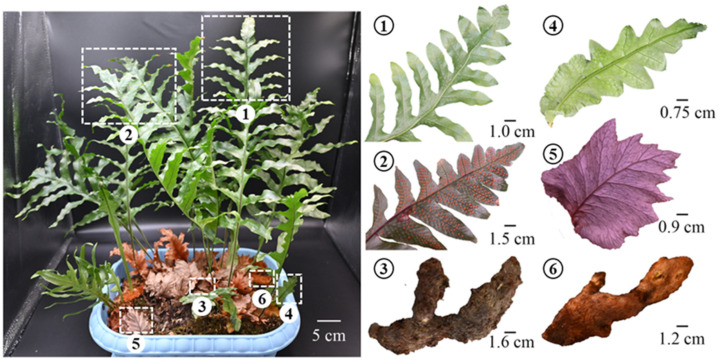
Morphological characteristics of different botanical parts on *D. roosii*. (①—sporotrophophyll leaves with immature sporangium, ②—sporotrophophyll leaves with mature sporangium, ③—old rhizomes with black tomentum connected nest leaves, ④—young nest leaf, ⑤—old nest leaf, ⑥—young rhizomes with gold tomentum connected nest leaves).

**Table 1:** Comparison results of respiration and fluorescence parameters of SLs and NLs on *D. roosii*.

Parameters	SLs	NLs	*p* Value
Transpiration rate (E, mol·m^−2^·s^−1^)	0.0003 ± 0.0002	0.0001 ± 0.0001	0.190
Net photosynthetic rate (Pn, µmol·m^−2^ s^−1^)	1.0039 ± 0.2687	0.3063 ± 0.1068 **	0.001
Intercellular CO_2_ concentration (Ci, µmol mol^−1^)	262.4504 ± 62.4487	325.115 ± 50.7267	0.135
Stomatal conductance to water vapor (GSW, mol·m^−2^·s^−1^)	0.0165 ± 0.0109	0.0108 ± 0.0065	0.379
Stomatal conductance to boundary layer water vapor (GBW, m^−2^·s^−1^)	3.0215 ± 0.005	3.0508 ± 0.0014 **	0.000
Total conductance to water vapor (GTW, mol·m^−2^·s^−1^)	0.0164 ± 0.0108	0.0108 ± 0.0065	0.379
Total conductance to CO_2_ (GTC, mol m^−2^·s^−1^)	0.0103 ± 0.0068	0.0067 ± 0.0041	0.379
Vapor pressure deficit at leaf surface (VPD, kPa)	1.6554 ± 0.0132	1.2694 ± 0.0229 **	0.000
Steady state fluorescence under light (Fs)	256.2163 ± 24.5645	336.4477 ± 15.4555 **	0.000
Maximum fluorescence under light (Fm′)	913.6633 ± 114.7845	988.939 ± 58.948	0.267
Actual quantum yield of photosystem II photochemistry (PhiPS2)	0.7175 ± 0.0279	0.6591 ± 0.021 **	0.008
Electron transport rate (ETR, µmol m^−2^ s^−1^)	11.1807 ± 0.4356	8.345 ± 0.2614	0.000
Quantum efficiency of CO_2_ assimilation (PhiCO_2_, µmol·µmol^−1^)	0.0442 ± 0.0086	−0.2962 ± 0.004 **	0.000
The difference between maximum fluorescence and minimum fluorescence under light (Fv′)	0.7865 ± 0.0179	1.0009 ± 0.0001 **	0.000
Minimum initial fluorescence (Fo)	191.3968 ± 40.6225	277.0105 ± 22.5607 **	0.006
Maximum fluorescence after dark adaption (Fm)	1025.38 ± 236.7404	1203.0767 ± 54.1949	0.236
Maximum photochemical efficiency of PSII reaction centers when fully open under dark adaptation (Fv/Fm)	0.8105 ± 0.0272	0.77 ± 0.0086	0.031
Net photosynthetic rate under dark adaptation (Adark, µmol·m^−2^·s^−1^)	−0.5237 ± 0.1861	−0.7954 ± 0.2911	0.074

** means significant difference with *p* < 0.01.

**Table 2:** Comparison results of relative parameters calculated from response curves of SLs and NLs on *D. roosii*.

Curve Type	Fitting Parameters	SLs	NLs
Photosynthetic light response curve	Dark respiration rate	0.5284	0.7224
	Maximum net photosynthetic rate (Pnmax, µmol·m^−2^ s^−1^)	2.5738	0.4315
	Light saturation point (LSP, µmol·m^−2^·s^−1^)	467.7510	206.8240
	Light compensation point (LCP, µmol·m^−2^·s^−1^)	11.5740	16.8850
	Apparent quantum yield (AQY)	0.0117	0.0042
	Fitting residuals	1.7085	0.1802
	Determination coefficient	0.8364	0.8682
Photosynthetic CO_2_ response curve	Dark respiration rate	4.0670	1.3667
	Maximum net photosynthetic rate (Pnmax, µmol·m^−2^ s^−1^)	3.4172	−1.1921
	Saturation intercellular CO_2_ concentration (SIC, µmol mol^−1^)	1125.4500	343.0470
	CO_2_ compensation point (CCP, µmol mol^−1^)	250.5410	460.9870
	Fitting residuals	8.8957	0.6672
	Determination coefficient	0.7960	0.9110
	Dark respiration rate	4.0670	1.3667

## Data Availability

The original contributions presented in this study are included in the article/Supplementary Material. Further inquiries can be directed to the corresponding author(s).

## Supplementary Materials

The following supporting information can be downloaded at: https://www.mdpi.com/article/10.3390/metabo15120805/s1. Figure S1: The spatial distribution of 64 flavonoid components in ORs and NRs of *D. roosii*; Table S1: Statistical results of quality control of sequencing data of SLs and NLs of *D. roosii*; Table S2: Mapping results of sequencing data of SLs and NLs of *D. roosii*; Table S3: The expression and annotation results of DEGs of SLs and NLs of *D. roosii*; Table S4: The detailed metabolites profiles of *D. roosii*.
