# Supplementary material for: Functional Dimorphism Analysis of Sporotrophophyll Leaves and Nest Leaves of Drynaria roosii with Their Connected Rhizomes Based on Multi-Omics Analysis
Source: Metabolites. 2025 Dec 18;15(12):805. doi: 10.3390/metabo15120805 (PMC12734675; doi:10.3390/metabo15120805)
Supplement: Supplementary file 1 [file metabolites-15-00805-s001.zip › Supplementary materials.pdf]

Functional Dimorphism Analysis of Sporotrophophyll Leaves and Nest Leaves of *Drynaria roosii* with Their Connected Rhizomes Based on Multi-Omics Analysis

Ye Cao [1,2], Yan Ren [1,2], Yanlei Han [1], Xiaoqing Wang [3], Hui Li [1,2,4], Yong Zeng [5], Xiwen Li [1,6,*] and Ye Wang [1,2,*]

1 Jiangxi Key Laboratory for Sustainable Utilization of Chinese Materia Medica Resources, Institute of Traditional Chinese Medicine Health Industry, China Academy of Chinese Medical Sciences, Nanchang 330115, China
2 Jiangxi Institute of Traditional Chinese Medicine Health Industry, Nanchang 330115, China
3 Jiangxi Provincial Institute of Traditional Chinese Medicine, Nanchang 330046, China
4 Institute of Chinese Materia Medica, China Academy of Chinese Medical Sciences, Beijing 100700, China
5 School of Pharmacy, Chengdu University of Traditional Chinese Medicine, Chengdu 611137, China
6 Institute of Medicinal Plant Development, Chinese Academy of Medical Sciences, Peking Union Medical College, Beijing 100193, China
* Correspondence: ywang@itcmhi.ac.cn (Y.W.); xwli@icmm.ac.cn (X.L.)

Figure S1 The spatial distribution of 64 flavonoids components in ORs and NRs of *D. roosii*.

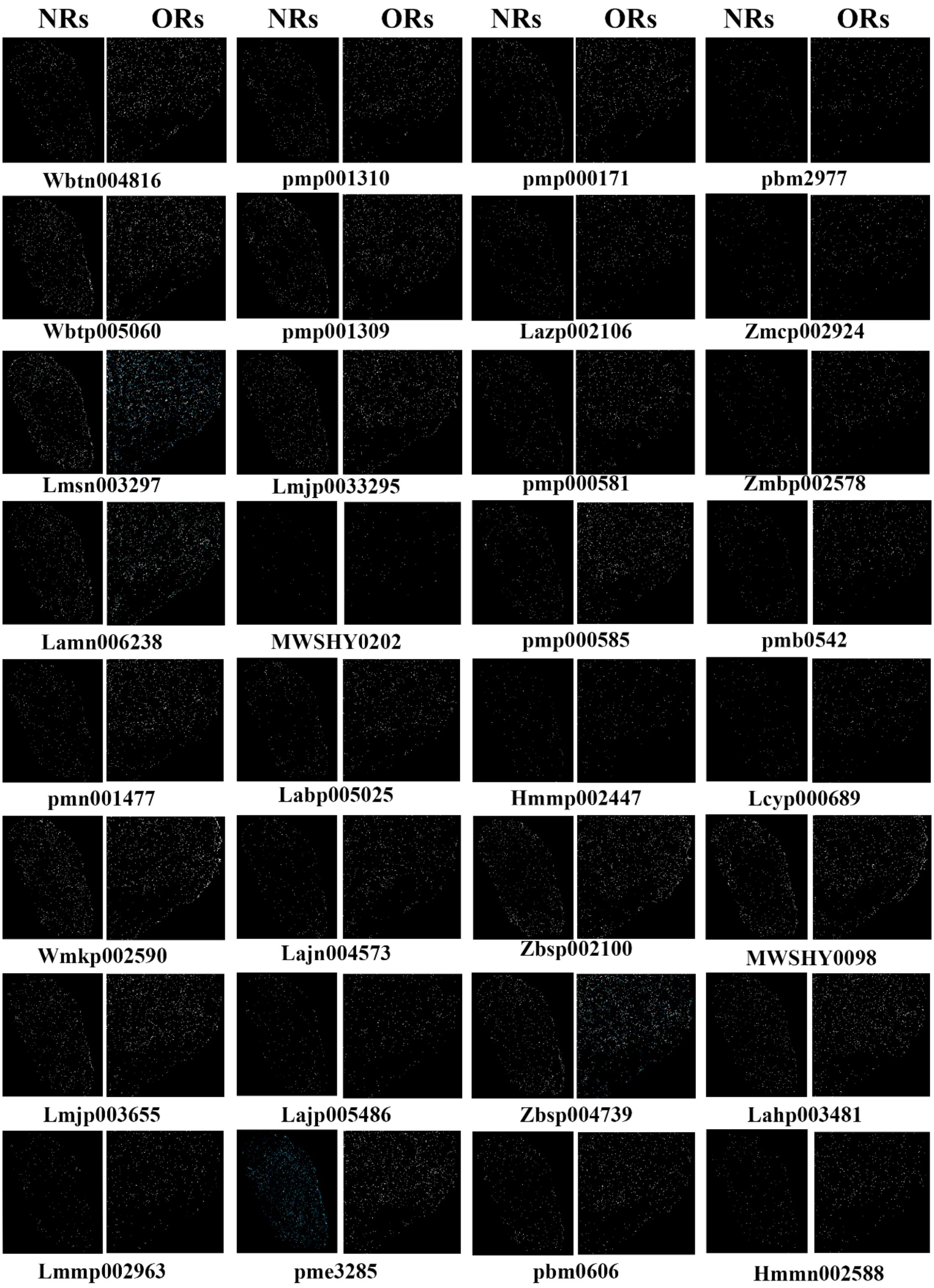

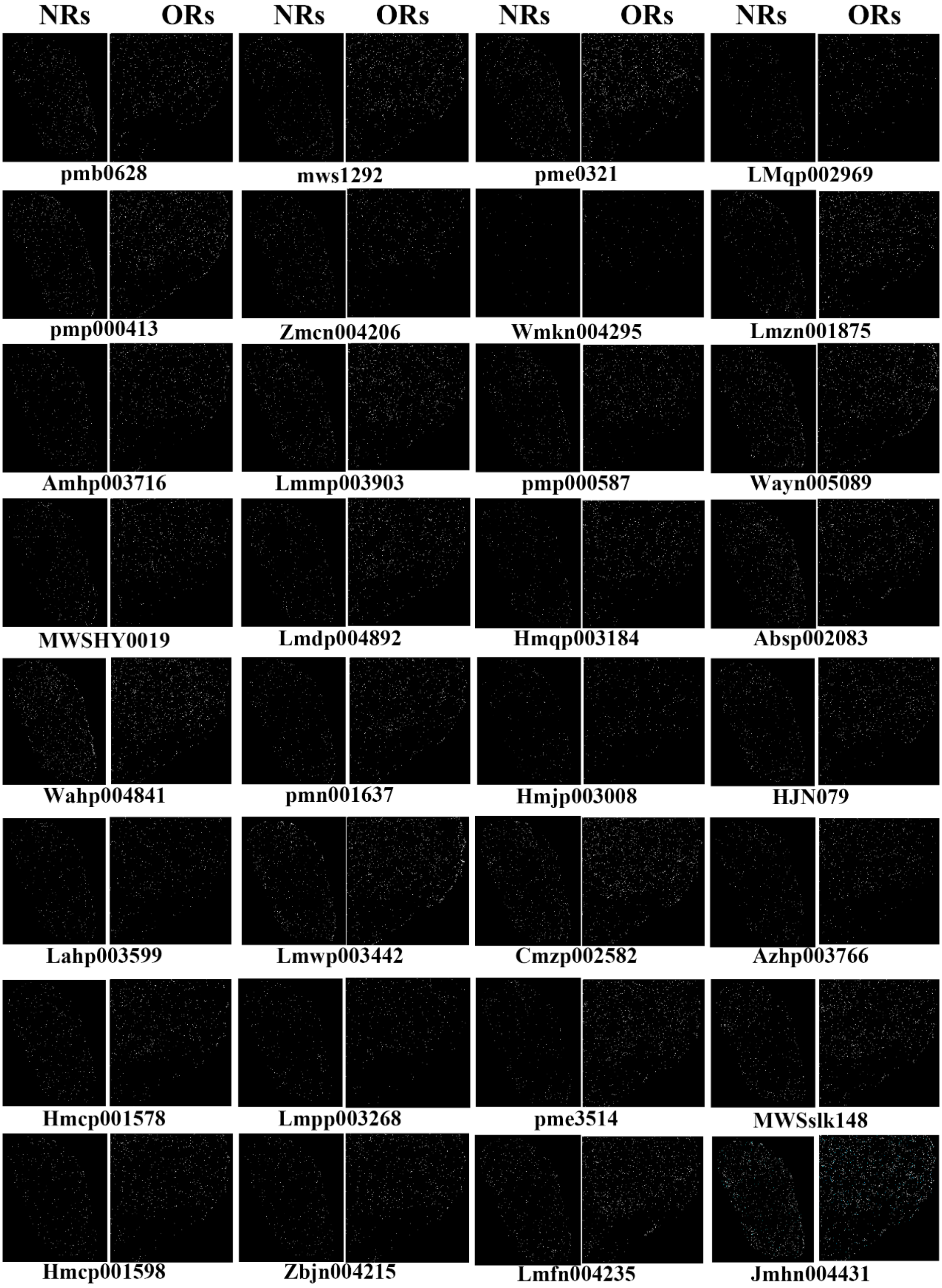
NRs
ORs
NRs
ORs
NRs
ORs
NRs
ORs
pmb0628
mws1292
pme0321
LMqp002969
pmp000413
Zmcn004206
Wmkn004295
Lmzn001875
Amhp003716
Lmmp003903
pmp000587
Wayn005089
MWSHY0019
Lmdp004892
Hmqp003184
Absp002083
Wahp004841
pmn001637
Hmjp003008
HJN079
Lahp003599
Lmwp003442
Cmzp002582
Azhp003766
Hmcp001578
Lmpp003268
pme3514
MWSslk148
Hmcp001598
Zbjn004215
Lmfn004235
Jmhn004431

Table S1 Statistical results of quality control of sequencing data of SLs and NLs on the *D. roosii*.

Sample	Raw reads	Raw bases	Clean reads	Clean bases	Clean ratio	Q20	Q30	GC
NL_1	40719400	6107910000	40429050	6008075698	99.29%	98.29%	94.94%	47.12%
NL_2	40985798	6147869700	40722744	6031619972	99.36%	98.43%	95.28%	46.79%
NL_3	40767050	6115057500	40510928	5913912679	99.37%	98.54%	95.55%	46.11%
SL_1	41130666	6169599900	40869342	6056389777	99.36%	98.43%	95.24%	46.09%
SL_2	41819434	6272915100	41538090	6192381618	99.33%	98.43%	95.20%	47.26%
SL_3	41637060	6245559000	41350790	6130355439	99.31%	98.33%	95.01%	46.42%

Table S2 Mapping results of sequencing data of SLs and NLs on the *D. roosii*.

Sample	Total reads	Total mapped reads	Unique match	Multi-position match	Percent of mapped reads
NL_1	40429050	36616160	8411350	28204810	90.57%
NL_2	40722744	37203572	8624224	28579348	91.36%
NL_3	40510928	37583858	8071730	29512128	92.77%
SL_1	40869342	37613372	9068718	28544654	92.03%
SL_2	41538090	37666900	9013250	28653650	90.68%
SL_3	41350790	37854136	9025852	28828284	91.54%
